# Ex Vivo Histological Analysis of Corneas with Manually Implanted Intracorneal Stromal Ring Segments

**DOI:** 10.3390/jcm13113350

**Published:** 2024-06-06

**Authors:** Noa Kapelushnik, Liliana Werner, Nadav Levinger, Samuel Levinger, Irina S. Barequet

**Affiliations:** 1 Goldschleger Eye Institute, Sheba Medical Center, Sackler Faculty of Medicine, Tel Aviv University, Tel Aviv-Yafo 6997801, Israel; kapelushniknoa@gmail.com; 2 John A. Moran Eye Center, University of Utah, Salt Lake City, UT 84112, USA; 3 Enaim Refractive Surgery Center, Jerusalem 9438307, Israeldr.ronin@enaim.co.il (S.L.)

**Keywords:** ring segments, corneal deposits, histology

## Abstract

**Backgrond:** Intracorneal ring segments (ICRSs) are utilized to correct refractive changes impacting visual acuity, commonly implanted via femtosecond laser but can also inserted manually. Corneal deposits alongside the ICRS channels are seen commonly. **Methods:** This study explores the histological characteristics of corneal deposits following manual ICRS implantation, comparing them to previously published articles describing femtosecond laser-assisted cases. **Results:** This is a retrospective analysis of three cases involving manual ICRS implantation, accumulation of whitish deposits and later explanation of the corneas due to penetrating keratoplasty (PKP). Patient demographics, ocular history, and surgical details were collected. Histological analysis employed Hematoxylin and Eosin (H&E) and Masson’s trichrome staining. Whitish deposits along ICRS tracts were observed in all cases, with minimal fibroblastic transformation of keratocytes adjacent to the segments. Comparing these cases of manual to femtosecond laser-assisted ICRS implantation, in most cases, similar deposits were identified, indicating the deposits’ association with the stromal tissue reaction to the ring segment and not to the surgical technique. **Conclusions:** This study contributes insights into the histopathology of manually implanted ICRS, emphasizing the shared nature of deposits in both insertion methods. The findings highlight the link between deposits and the stromal tissue reaction to the ring segment, irrespective of the insertion technique.

## 1. Introduction

Intracorneal ring segments (ICRS) are devices implanted in the corneal stroma, to correct refractive changes causing a decrease in visual acuity. The inception of intracorneal rings can be traced back to Reynolds in 1978 when he introduced a 360-degree ring-shaped device designed to modify the anterior corneal curvature, aiming to flatten the cornea and address myopia. The distinct advantages of intracorneal rings, compared to alternative refractive surgeries, include the preservation of the cornea’s prolate structure and a reduction in corneal wound impact. The proposed mechanism involves the reshaping and flattening of the cornea through the influence of the inserted ring on the cornea’s collagen fibrils. Initial human studies validated the safety of intracorneal ring insertion, demonstrating successful corneal flattening to alleviate myopia. Importantly, this corneal effect was reversible, as the removal of the device led to a return to the preoperative corneal state. Subsequent modifications resulted in the transformation of the device into two separate arcs, now recognized as intracorneal ring segments (ICRS) [1]. ICRS are mostly crescent-shaped devices made from a biocompatible material such as polymethyl methacrylate (PMMA). The implantation procedure of ICRS involves creating a small incision and tunnels in the cornea, previously manually but in recent years by femtosecond laser, and inserting the segments into the corneal stroma. In manual or mechanical insertion of ICRS, the center of the cornea is marked using a surgical marker. Using a diamond knife set to 70% of the corneal thickness at the incision site, a vertical incision is made into the cornea. From this incision, corneal pockets are meticulously fashioned on each side, ensuring uniform depth across the entire width within the same stromal plane and extending as far as the stromal spreader. The cornea is stabled using a vacuum centering guide. The glide is inserted into the corneal pockets, and the dissector blade is rotated beneath it to create the intrastromal tunnels. A single intrastromal corneal ring segment is inserted into each tunnel, leaving the positioning hole 1 to 2 mm from the incision site. Interrupted sutures are placed evenly to ensure secure wound closure, with suture depth matching the level of the stromal pocket to prevent segment migration. Suture knots are buried, and postoperative administration of antibiotic-corticosteroid eye drops is initiated for at least a week, therapeutic contact lenses are usually placed. In femtosecond laser-assisted insertion of ICRS, the tunnels are created using the laser with selected parameters. Usually, they have a depth of 400 µm, an incision width of 1 mm, and a length of 1.4 mm. After the creation of the tunnel by the laser the lip of the incision is identified using a Sinsky hook, and then the segment ring is placed in the tunnel [2]. Once in place, the segments exert mechanical forces on the cornea, flattening the central area and improving its optical properties. This helps to correct myopia, reduce astigmatism, and improve overall visual quality. In Refs. [2,3], ICRSs are also used for the treatment of keratoconus as first published more than 20 years ago. In Ref. [3], it is also used for the treatment of corneal ectasia and more specifically post-laser in situ keratomileusis (LASIK) ectasia [4]. Several complications related to ICRS insertion were reported; the common postoperative complications are ring extrusion, migration, corneal thinning and infective keratitis [5]. Ring extrusion is the late complication of ring migration and corneal thinning. Wrong implantation of the ICRS, usually shallow, causes increased strain which can lead to stromal breakdown, corneal thinning and eventually ring extrusion. Infective keratitis following ICRS implantation is rare but has the potential to cause vision loss. The sterile environment of the surgery reduces the chances of infectious keratitis. These complications together with subjective complaints of visual disturbances such as glare, halos, fluctuating vision and complaints of pain and foreign body sensation, are the main cause of ICRS extraction. Another complication involves intrastromal deposits near the ICRS. The structural and refractive implications of these deposits remain uncertain [6]. Because the deposits primarily exist adjacent to the ring segments channel and do not affect the central area of the cornea, it is believed that they have no impact on vision [7], but since the deposits might be related to an inflammatory reaction of the corneal stroma, they could be related to anterior stromal necrosis [8].

The aim of this study is to present cases of intrastromal corneal deposits after manual implantation of ICRS. In all cases, the corneas containing the ICRS were extracted due to PKP carried out for vision rehabilitation. The histological characteristics of the cases are compared to previous published histological analyses of corneal deposits after femtosecond laser-assisted ICRS implantation.

## 2. Methods

This is a retrospective study of corneal histological analysis of corneal buttons containing ICRS with visible corneal deposits around the ICRS. All patients underwent l penetrating keratoplasty (PKP) for visual rehabilitation and had a prior history of ICRS implantation via manually created channels. The patients were operated on, treated, and evaluated in a single center by a single surgeon (SL). Data including baseline demographics and ocular history were collected. This research adhered to the tenets of the Declaration of Helsinki, and Institutional Review Board (IRB) approval was obtained. Patient consent was waived for this anonymized retrospective study.

### 2.1. Surgical Technique (ICRS)

All patients had Intacs^®^ (Addition Technology Inc., AJL Ophthalmic, S.A., Gasteiz, Spain) inserted with an arc thickness of 0.25–0.35 mm, an external diameter size of 8 mm and an internal diameter size of 6.8 mm. ICRS was manually dissected via channels and under topical anesthesia (Localin; Oxybuprocaine hydrochloride 0.4%; Fischer Labs Ltd., Tel Aviv, Israel). The center of the cornea was marked to ensure the exact site of incision. Pachymetry was measured at the peripheral location of the entry incision. A 1 mm radial incision was performed at a depth of 75% of the corneal thickness with a calibrated diamond knife. Intrastromal pockets were created using pocket micro dissectors. Using a guide, the channel dissector was inserted into the pockets and a tunnel was created. The ring segment was then inserted into the channel using a guide and a Sinskey hook. At the end of the procedure, a bandage contact lens (Purevision, Bausch & Lomb, Inc., Bridgewater, NJ, USA) was placed following the application of Dexamethasone Sodium Phosphate 0.1% and Moxifloxacin Hydrochloride 0.5%. The postoperative regimen included Moxifloxacin Hydrochloride 0.5% and Dexamethasone Sodium Phosphate 0.1% 4 times a day until the removal of the bandage contact lens.

### 2.2. Histological Analysis

Ex vivo histological analysis of the excised cornea buttons containing the ICRS was performed. All corneas were fixed in 4% formaldehyde and forwarded to the John A. Moran Eye Center, University of Utah for complete histopathological assessment. Once received in the laboratory, the specimens were fixated in 10% neutral buffered formalin for 24 h. Gross examination was performed, and gross pictures were taken using a Nikon digital camera (model D1x with a Nikon ED 28–70 mm AF lens). Each cornea containing the ICRS underwent complete histopathological processing. After embedding in paraffin, 5-micron-thick histopathological sections passing through the center of the corneas containing the ICRS were cut and stained with hematoxylin and eosin (H&E) and Masson’s trichrome stains. Hematoxylin stains nuclear components, including heterochromatin and nucleoli in dark purple, while eosin stains cytoplasmic components including collagen and elastic fibers, muscle fibers and red blood cells in pink. Masson’s trichrome is a tri-color stain used to distinguish cells from connective tissue. It stains collagen in blue, nuclei in dark red/purple, and muscle as well as cytoplasm in red. Microscopic examination and photographs (taken at different magnifications) of the different sections were completed under a light microscope (Olympus, Optical Co., Ltd., Tokyo, Japan).

## 3. Results

Three corneas of three patients were included in this study. Two are males and one female. The insertion of the Intacs^®^ Corneal Implants was performed in channels created manually. The operations went unremarkably in all cases with no intraoperative complications. The reason for ICRS implantation was keratoconus in one patient and post-LASIK ectasia in the other two. In all three patients, significant whitish dotted deposits spread along the segments’ tracts were observed. All these patients required PKP for visual rehabilitation due to unsatisfying vision. During the PKP the corneas including the ICRS were removed at 9–12 months after the initial ICRS implantation. The ages of patients at the time of PKP were 23, 40 and 28 years, respectively. The first patient underwent LASIK at the age of 20 and developed ectasia in his left eye. ICRSs were implanted using the manual technique. His visual rehabilitation was limited; therefore, he underwent a PKP a year later, which included the ICRS in the explanted tissue. The second patient underwent LASIK at the age of 36. She developed ectasia in her left eye 3 years later and ICRSs were implanted using the manual technique. However, she was not satisfied with the vision achieved and therefore a PKP was performed 9 months later, with the ICRS included in the explanted tissue. The third patient was diagnosed with keratoconus, and at the age of 27, underwent manual insertion of ICRS; a year later, they underwent PKP with the ICRS included in the explanted tissue. Patient characteristics are summarized in Table 1.

**Figure 1:**
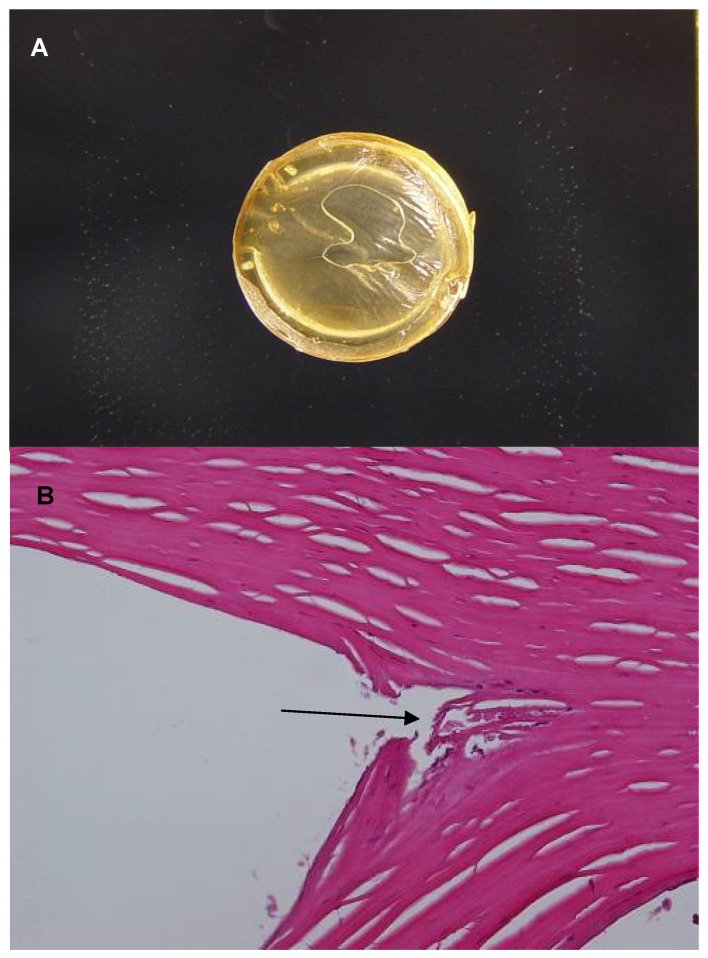
Patient 1. (**A**) Gross photographs of the cornea. Whitish deposits are seen at the inner curvature of the ICRS. (**B**) Light photomicrographs (H&E stain; original magnification ×400). The arrow points to a minimal fibroblastic transformation of keratocytes in contact with the segment.

**Figure 2:**
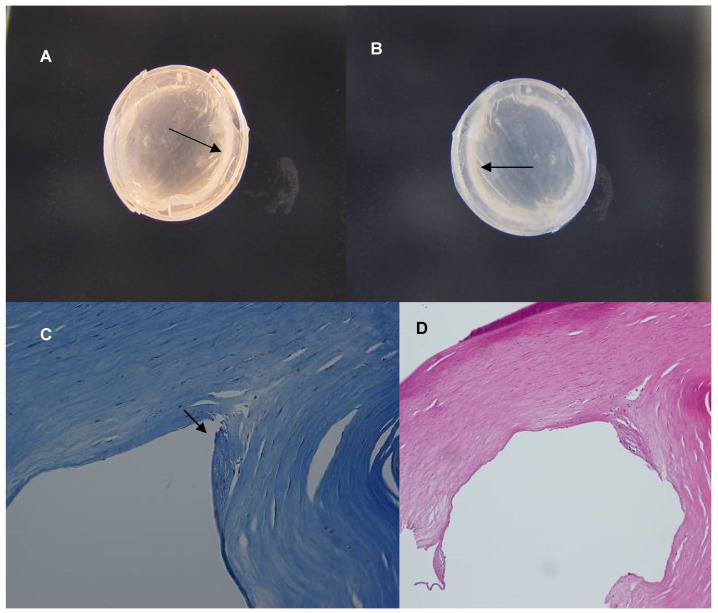
Patient 2. (**A**,**B**) Gross photographs of the cornea; (**A**) is the posterior view and (**B**) is the anterior view. Arrows point to white deposits next to the inner curvature of the ICRS. (**C**) Light photomicrograph, Masson’s trichrome stain, original magnification ×200; the arrow points to minimal fibroblastic transformation of keratocytes in contact with the segment. (**D**) Light photomicrograph, H&E stain, original magnification ×100. Same findings as in (**C**) are observed in the area in contact with the segment.

**Figure 3:**
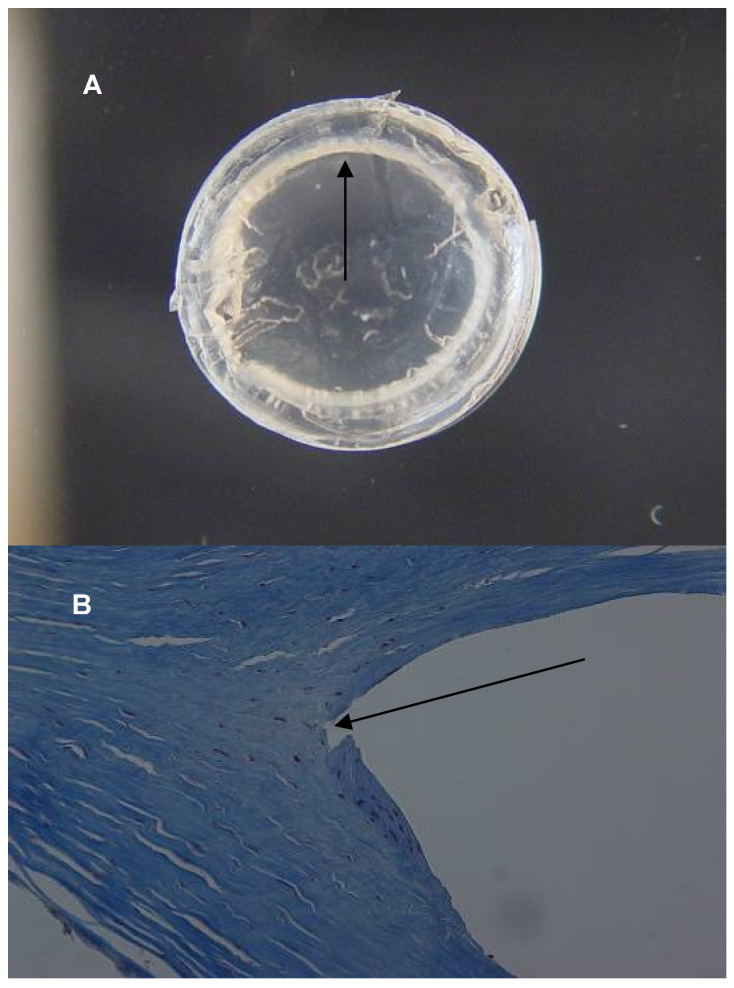
Patient 3. (**A**) Gross photograph of the cornea; the arrow points to white deposits next to the inner curvature of the ICRS. (**B**) Masson’s trichrome stain, original magnification ×200; the area in contact with the segment shows minimal fibroblastic transformation of keratocytes (arrow).

Macro pictures (gross photographs) and light photomicrographs from the histology analysis are presented in Figure 1, Figure 2 and Figure 3. In all the three cases, whitish deposits were present along the inner curvature of the ICRS. The area within the corneal stroma occupied by the ICRS appeared as an empty space in the histopathological sections, as the segments melted during histopathological preparation. In all cases, minimal fibroblastic transformation of keratocytes was observed in the areas that were in contact with the segment.

## 4. Discussion

This study outlines three instances where corneas underwent ICRS mechanical implantation followed by PKP. Each case exhibited clinical related to ICRS. Subsequently, all three corneas housing the ICRS were excised during the PKP and were subjected to histological examination using light microscopy. The analysis revealed whitish deposits situated alongside the inner curvature of the segments. There was an absence of inflammatory reactions, and minimal fibroblastic transformation of keratocytes was observed in the area adjacent to the segments. The nature of the deposits has been previously investigated [9,10,11,12,13]; in this research, we described our cases in which the ICRS was manually inserted.

For more than thirty years, ICRSs have been clinically used for visual improvement in different refractive and corneal diseases [14]. Initially, the common practice was to manually create the channel in which the ring segments were implanted. Ultrafast pulse lasers have been developed to reduce the energy required for tissue incision and minimize damage to surrounding tissues. The IntraLase femtosecond laser received FDA approval for lamellar corneal surgery in 2004. This laser uses pulses as short as 10–15 s. It employs an infrared wavelength of 1053 nm, the laser produces a scanning pulse focused to 3 μm with an exceptional accuracy of 1 micrometer. The laser is able to cut a precise spiral pattern in the corneal stroma and thus is used in intrastromal surgeries [15]. In recent years, the use of a femtosecond laser to assist with the tunnel creation has been more common [16]. Since the laser allows the surgeon to create a more accurate tunnel for the ICRS, it theoretically has the potential to bring better optical results. Pinero et al. compared visual and refractive results of manually inserted and femtosecond laser-assisted insertion of ICRS. After two years of follow-up, the refractive and visual outcomes of both surgical methods were similar [17]. Although the visual outcome seems to be similar in femtosecond laser-assisted ICRS and manual insertion of ICRS, the use of a femtosecond laser has many benefits such as less tissue stress and lower complication rates, as concluded by Struckmeier et al. In the meta-analysis which included 115 studies, mechanical creation of the stromal tunnel in ICRS is associated with higher complication rates compared to femtosecond laser-assisted tunnel creation [9]. One of the post-operative complications seen in ICRS implantation is the accumulation of stromal deposits next to the ICRS. The deposits are very frequent and seen in animal models as well. Parks et al. used hydrogel intrastromal lenticules in primates. Intrastromal deposits were noted adjacent to the lenticule. These deposits were found to be lipid crystals. They concluded that stromal keratocytes produce lipids as a response to stress when inserting an intrastromal lenticule—most probably mechanical stress [10]. Twa et al. described the histological changes associated with the manual insertion of ICRS in New Zealand white rabbits. The observed deposits were characterized as intracellular saturated lipid material [11]. Ruckhofer et al. reviewed two years of follow-up data from 359 patients from 10 different investigational sites who were implanted with ICRS. Two years postoperatively, 74% of the corneas had channel deposits, most of them located along the inner curvature of the segment, similar to the cases presented in this study. Their study lacks a description of the surgical procedure, so it is unclear whether the ICRS channels were created mechanically or using a femtosecond laser [12]. The nature of the deposits was investigated by Ruckhofer et al., who used in vivo confocal microscopy to characterize the deposits. In their study, the deposits were described as a highly reflective material, with amorphous aggregation and no definite cellular features. Similar to this study, mild fibrosis and no sign of inflammation were seen [12]. Hamon et al. analyzed the stromal tissue changes after femtosecond laser-assisted ICRS implantation. Their study included 15 patients; the authors recovered two explanted recipient corneal tissues with implanted ICRS segments for analysis. The first explanted cornea was fixed in neutral buffered 4% formaldehyde and processed for histopathological analysis (H&E and Masson’s trichrome stains). The second explanted cornea was fixed in 3% cacodylate-buffered glutaraldehyde and processed for transmission electron microscopic (TEM) analysis. Like our results, histopathology showed a proliferation of fibroblasts with mild fibrosis [6]. TEM of the second explanted cornea showed peri-segmental fibrotic stromal changes within a narrow zone of 3 to 15 µm. Within this zone, amorphous and vacuolar materials were found to be interspersed between the condensed collagen fibers. Few degenerative keratocytes containing cytoplasmic lipid inclusions were occasionally observed in this zone. In regions of clinical lamellar channel deposits, focal accumulations of degenerative keratocytes containing large amounts of cytoplasmic lipid inclusions could be visualized. As expected, no lipids could be visualized under histopathology as these were dissolved during the preparation process. In this study, the extracted corneas were fully processed for complete standard histopathological analysis, which, as mentioned above, resulted in the lipid melting. Recognition of lipid accumulation in formalin fixed paraffin imbedded specimens is a challenge, and new techniques are recently being developed to support pathologist in the recognition of lipids [13]. One example is a study by Harada et al., which proposed a new method for detection lipid droplets in pathological images using reinforcement learning. Other methods for visualizing lipid depositions include using labeling the lipids with specific probes and dyes such as Oil Red O or using special microscopic techniques such as differential interference microscopy, coherent Raman microscopy, transmission electron microscopy or scanning electron microscopy. These methods are not routinely used, but according to this study, in future studies, it could be advised to use such methods to better elucidate the nature of the ICRS deposits more clearly.

In another case study conducted by Al-Amri et al., the occurrence of stromal deposits adjacent to the implanted intracorneal ring segments (ICRS) was documented in femtosecond laser-assisted ICRS channels. The case involved the insertion of a 450 µm thick intact segment in the cornea of a 27-year-old patient with keratoconus and contact lens intolerance. Five months post-segment insertion, the presence of whitish intra-stromal deposits was noted. Due to unsatisfactory visual improvement, penetrating keratoplasty was performed, and the excised corneas underwent histological analysis. The examination revealed the presence of intra-stromal foamy histiocytes surrounding the segments [18]. The presence of histiocytes in the analyzed deposits suggests that inflammatory response could be the etiology for the deposits seen next to the segments. In our cases, no foamy histiocytes were observed in the histopathological evaluation.

Femtosecond laser-assisted ICRS implantation has its advantages in causing less tissue stress, as mentioned above, but when it comes to ICRS deposits, it seems that there is not much difference in the nature of the deposits in manually inserted ICRS vs. femtosecond laser-assisted ICRS implantation. In one case, which described the presence of histiocytes adjacent to the segments, femtosecond laser-assisted ICRS implantation can suggest an inflammatory response mechanism for deposit formation. As we know, it is the only case describing such findings, so it is hard to correlate these histological findings to the segment insertion mechanism. It can be concluded that the deposits are secondary to the stromal tissue reaction to the ring segment itself and the tissue stress they cause rather than the surgical method used to insert them. In future studies, it is advisable to incorporate immunostaining for proliferative and inflammatory markers, along with techniques for enhanced visualization of lipid accumulation, into histological analysis.

The study has some limitations, such as its retrospective nature and the small number of corneas eventually included, though there are more than previously published. The patients enrolled in this study underwent PKP due to unsuccessful visual rehabilitation and dissatisfaction with their vision after ICRS implantation. It is important to note that PKP is not the sole treatment option for such cases. Non-surgical approaches for improving vision after ICRS insertion include the use of contact lenses, such as rigid gas permeable lenses, which can help mask corneal irregularities affecting vision [18]. Surgical options for addressing unsatisfactory vision after ICRS implantation typically involve ICRS extraction. Another possibility is photorefractive keratectomy combined with corneal cross-linking [19]. The low number of participants in this study is attributed to the rarity of patients who undergo PKP with ICRS still in place within the extracted cornea. The small sample size and the retrospective nature of the study contributed to the comparison method of the study. However, the small sample size of the study limits our ability to compare other factors that could influence the nature of the corneal deposits, such as the race, age, and gender of the patients. Future studies should be prospective and compare not to the published literature but to patients included in the study.

In conclusion, this study presented the histopathology of corneas with manually implanted intracorneal stromal ring segments, which were explanted during PKP. To our knowledge, the histopathology was previously described, but mostly regarding femtosecond-assisted ICRS implantation and not in the context of manually inserted segments as we did in this study. These data shed more light on the etiology of the accumulation of the deposits and its relation to the surgical procedure. The results of this study emphasize that the deposits are associated with the implant itself rather than the surgical procedure.

## Figures and Tables

**Table 1:** Patient clinical data.

Patient	Gender	Diagnosis	Age at ICRS Insertion (Years)	PKP Indication	Time from ICRS Insertion to PK (Years)
1	Male	Post LASIK ectasia	22	Limited visual rehabilitation	1
2	Female	Post LASIK ectasia	39	Limited visual rehabilitation	0.75
3	Male	Keratoconus	27	Unsatisfactory vision improvement	1

## Data Availability

Data available on request due to privacy restrictions.
